# A Fast, Stability-Indicating, and Validated Liquid Chromatography Method for the Purity Control of Lercanidipine Hydrochloride in Tablet Dosage Form

**DOI:** 10.3797/scipharm.1310-10

**Published:** 2014-01-16

**Authors:** Saumil Mehta, Sukhdev Singh, Kishor Chikhalia

**Affiliations:** 1 Torrent Research Centre, Bhat, Gandhinagar, Gujarat, India.; 2 Department of chemistry, school of sciences, Gujarat University, Ahmedabad, Gujarat, India.

**Keywords:** Lercanidipine, Impurities, RRLC, Stability indicating, Validation

## Abstract

A robust, sensitive, and stability-indicating rapid resolution liquid chromatography method for the simultaneous determination of process impurities and degradation products of lercanidipine hydrochloride in pharmaceutical dosage form was developed and validated. The chromatographic separation was performed on the Zorbax SB C18 [(50 × 4.6) mm] 1.8 μm column, using gradient elution of a potassium dihydrogen phosphate buffer (pH 3.5, 0.01 M) and acetonitrile. The flow rate was 1.0 ml/min and UV detection was performed at 220 nm. The method was further evaluated for its stability-indicating capability by hydrolytic, oxidative, thermal, thermal with moisture, and photolytic degradation studies. All acceptance criteria of the International Conference on Harmonization guidelines for validation were covered in the method validation. This method can be used for purity control during manufacture and real time stability studies. A shorter run time of 10 minutes and good solution stability for at least 48 hours allowed the quantification of more than 50 samples per day with comparatively lower costs than existing methods.

## Introduction

**Fig. 1:**
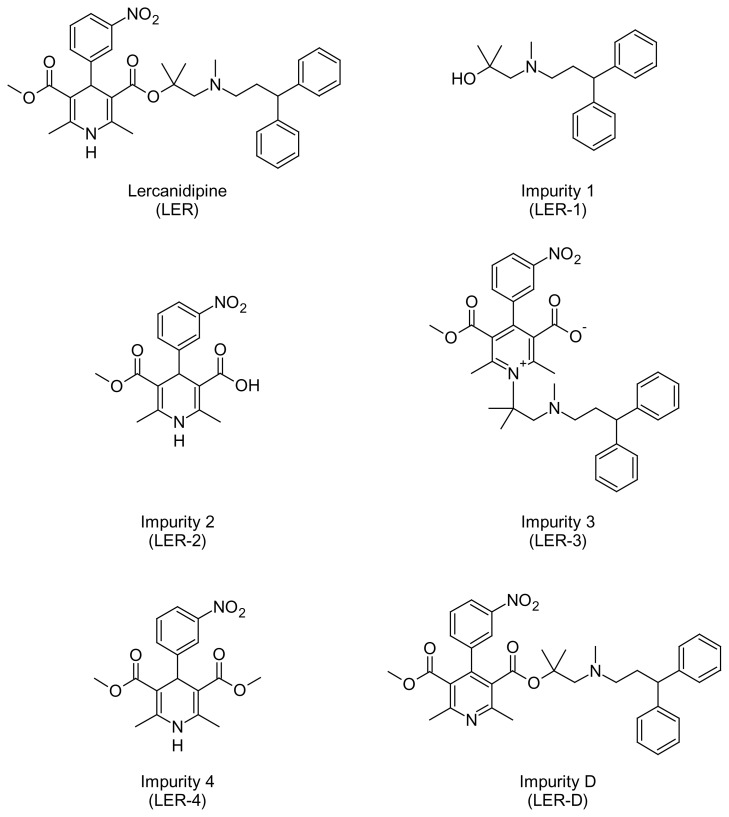
Structure of lercanidipine (LER) and its impurities LER-1–4, and LER-D

Lercanidipine is a dihydropyridine calcium-channel blocker used in the treatment of hypertension. It is given orally as the hydrochloride form in an initial dose of 10 mg once daily before food, increased if necessary after at least two weeks to 20 mg daily. Chemically, lercanidipine hydrochloride (LER) is (±)-2-[(3,3-diphenylpropyl)(methyl)amino]-1,1-dimethylethyl methyl 2,6-dimethyl-4-(3-nitrophenyl)-1,4-dihydropyridine-3,5-dicarboxylate hydrochloride [1]. The chemical structure of LER is given in Fig. 1.

LER is not an official drug in any pharmacopoeia. Several analytical methods such as spectrophotometric [2–7], voltametric [8], and HPLC [9–11] methods are reported for the estimation of LER in bulk drugs and formulations. The literature survey revealed that two HPLC [12–14] methods were reported for the estimation of LER and its process impurities in the formulation. An investigation on the photochemical stability of LER and its determination in tablets by HPLC-UV and LC–ESI–MS/MS had also been studied [15]. As per our knowledge, no method for separation of degradation products and no force degradation study except photochemical stability had been reported in any method.

Rapid resolution liquid chromatography (RRLC) is a technique in liquid chromatography which enables a reduction in separation time and solvent consumption without compromising resolution power. The purpose of this study was to develop a stability-indicating RRLC method, which has not yet been published for the estimation of degradation products and five known related compounds [Lercanidipine impurity 1 (LER-1), Lercanidipine impurity 2 (LER-2), Lercanidipine impurity 3 (LER-3), Lercanidipine impurity 4 (LER-4), and Lercanidipine impurity D (LER-D)] and its major degradation products in tablets. LER-1, LER-2, LER-3, and LER-4 are process impurities, while LER-D is a process impurity as well as a degradation impurity. The chemical structures of LER-1, LER-2, LER-3, LER-4, and LER-D are given in Fig. 1. Thereafter, this method was validated and successfully applied for the separation and quantification of all compounds of interest in the pharmaceutical formulation.

## Experimental

### Chemicals and Reagents

Reference standards of LER, LER-1, LER-2, LER-3, LER-4, and of LER-D were with purities of 99.79%, 97.23%, 95.46%, 98.26%, 98.84%, and 97.36%, respectively. Tablet formulations containing 10 mg of LER were prepared in the laboratory as a process of developing the finished product. All reference standard and formulation samples were provided by Torrent Research Centre, Gujarat, India.

HPLC grade acetonitrile (Rankem, India), methanol (Rankem, India), analytical reagent grade orthophosphoric acid (Rankem, India), potassium dihydrogen phosphate (Rankem, India), and triethylamine (Rankem, India) were used. The nylon filters with a pore size of 0.22 μm (Waters, Milford, USA) were used to filter solutions.

### Preparation of Buffer

A solution of potassium dihydrogen phosphate (0.01 M) and triethylamine (0.1%, v/v) was prepared by dissolving 1.36 g of potassium dihydrogen phosphate and 1 ml of triethylamine in one liter of water for HPLC. The pH of this solution was adjusted to 3.5±0.02 with orthophosphoric acid. This solution was then filtered through a 0.22 μm nylon filter. The buffer preparation was found to be stable with respect to pH and visual clarity for about 50 hours.

### Chromatographic Parameters

Analysis was performed on the 1200 SL system (Agilent, USA), consisting of a binary solvent manager, autosampler manager, and PDA detector. The output signal was monitored and processed by Chemstation software. Separation was carried out on a Zorbax SB C18 column [(50 × 4.6) mm], 1.8 μm (Agilent, USA). The separation was achieved by gradient elution (Table 1) with a flow rate of 1.0 ml/minute. The detection was monitored at a wavelength of 220 nm. The column temperature was maintained at 50°C and the injection volume was 5 μl.

### Standard Solution Preparation

Standard solution was prepared by dissolving the reference standard in methanol to obtain the concentration of 0.5 μg/ml of LER, LER-1, LER-2, LER-3, LER-4, and LER-D.

### Sample Solution Preparation

Ten tablets were crushed to a fine powder. An accurately weighed portion of the powder equivalent to 25 mg of LER was taken in a 250-ml volumetric flask. Then 75 ml of methanol was added and it was sonicated for about 30 minutes with intermittent shaking. After cooling to ambient temperature, this solution was diluted up to the mark with methanol, mixed and filtered through a 0.22 μm nylon filter, and the filtrate was collected after discarding about 10 ml. The concentration of the sample preparation was 100 μg/ml.

## Method Validation

The method was validated to demonstrate that it is suitable for its intended purpose by the standard test procedure to evaluate adequate validation characteristics (specificity, accuracy, precision, linearity, LOD, LOQ, solution stability, and robustness). Method validation covers all parameters and acceptance criteria defined in the International Conference Harmonization guidelines for analytical method validation [16].

### System Suitability

System suitability parameters were checked to verify the system performance by injecting the standard solution preparation. System precision was determined on three replicate injections of the standard solution preparation in which the relative standard deviation (RSD), theoretical plate number, and tailing factor were checked. The resolution between critical pairs was also checked.

### Specificity

Specificity studies were performed to demonstrate the selectivity and stability-indicating capacity of the proposed method. The selectivity and specificity of the method was studied by injecting the blank preparation (methanol), placebo preparation, impurity mixture preparation, and LER standard preparation. Forced degradation studies were performed on the LER formulation to measure the power of the stability-indicating property and specificity of the proposed method. The blank, placebo, LER standard, and powder sample of the tablets were exposed to acidic (1 N hydrochloric acid, 80°C, 60 minutes), alkaline (1 N sodium hydroxide, 50°C, 60 minutes), oxidizing (10% hydrogen peroxide, 50°C, 60 minutes), thermal (100°C, 6 hours), thermal moisture (spiked 10 water, 100°C, 6 hours), and photolytic (1.2 million lux hours) degradation conditions. All of these exposed preparations were analyzed by the proposed method on the photodiode array detector.

### Linearity

The linearity of the method was determined at various concentration levels (0.01, 0.03, 0.05, 0.1, 0.2, 0.5, 2, and 3 μg/ml) for LER and for each known impurity. The correlation graph was constructed by plotting the peak areas versus actual concentrations for each peak. The correlation coefficient, slope, and response factors were calculated.

### Limit of Detection (LOD) and Limit of Quantitation (LOQ)

A series of diluted solutions of known concentrations of LER and its impurities were injected to achieve the LOD and LOQ by the signal-to-noise ratio (S/N) method as per ICH guidelines. The %RSD values were determined for each peak by injecting six replicates at the LOQ concentration level.

### Precision

Method precision was examined by analyzing six preparations of the LER sample solution spiked with a mixture of impurities at the specification limit by the proposed method. The %RSD for each impurity value and the total impurities value were also determined. Intermediate precision was studied on a different day, using a different column, and on a different instrument.

### Accuracy

To perform the accuracy of the proposed method, recovery experiments were carried out by the standard addition technique. The accuracy of the method was evaluated in triplicate preparation at four different concentration levels – LOQ, 50%, 100%, and 150% of the target specification concentration (0.5 μg/ml). The percentage recoveries for LER and for the impurities at each level and for each replicate were calculated by the standard subtraction technique. In such a sample preparation, the LER-D was found to be 0.13%. This obtained value was subtracted in the final calculation for each recovery preparation. The mean of the percentage recoveries (n = 12) was calculated.

### Robustness

To determine the robustness of the analytical method, experimental conditions were deliberately altered. Changes in the chromatographic conditions were studied by testing the influence of small changes in the pH of the buffer (±0.02 units), and change in the column oven temperature (±5 units). The blank preparation, injections for system suitability, and the sample preparation were injected for the robustness study.

### Stability of the Standard Preparation Solution and Sample Preparation Solution

The stability of the standard solution and sample solution was established by storing the solutions for 48 hours at ambient temperature and at 15°C in light and in the dark. The stored solutions were re-analyzed after 12 hours, 24 hours, and 48 hours. The impurities were calculated against the standard preparation and compared against the fresh sample preparation.

## Results and Discussion

### Method Development and Optimization of Chromatographic Parameters

**Fig. 2:**
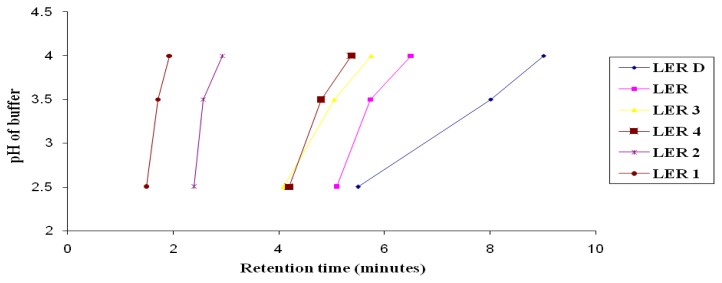
Effect of pH of buffer on retention of compounds

The method was started to develop on low dimensions with a lower particle sized column to achieve the goal of a shorter run time without compromising resolution. From the UV profiling, it was found that the suitable wavelength for the LER and its related impurities was 220 nm. As LER is basic with a pKa value of 9.36, trials were started and made with buffers having a pH of 2.5 or more. The Zorbax SB C18 [(50 × 4.6) mm], 1.8 μm column and potassium dihydrogen phosphate salt were selected for further development as they were the best choice for this pH range. Triethylamine was incorporated for better peak shape in the buffer preparation. Initially, when experiments were performed by using an isocratic mobile phase system with methanol instead of acetonitrile as the organic solvent in the mobile phase, late elution of the analyte peak and high pressure were observed. Hence, the experiments were carried out with acetonitrile as an organic solvent to develop a method with a shorter run time. To develop a stability-indicating method, the retention behaviour of these six compounds, with a change in percentage of acetonitrile and pH of the buffer, was studied on the selected column. While assessing the effect of change of the proportion of acetonitrile in the mobile phase, the pH of the buffer was constant at 2.5. Longer retention of all compounds was observed with a decrease in the percentage of acetonitrile in the mobile phase. While assessing the effect of the pH of the buffer, the mobile phase composition was kept as buffer-acetonitrile (60:40, v/v) and the retention of the compounds was plotted against the pH of the buffer preparation (Figure 2). This study showed that the retention of LER-D was highly dependent on the pH of the buffer. These studies showed that LER-4 and LER-3 were found to be co-eluting and LER and LER-D were found to be co-eluting next to them. LER-1 and LER-2 were relatively retained for a shorter time and were well-separated from all other compounds. Now, the main goal was to achieve better resolution between (1) LER-4 and LER-3 and (2) LER and LER-D. Different experiments indicated that the separation between LER-4 and LER-3 could be enhanced with a decrease in percentage of acetonitrile in the mobile phase. Fig. 2 indicates that separation between LER and LER-D enhances with an increase in the pH of the buffer. To achieve successful separation between LER-4 and LER-3, and between LER and LER-D, it was decided to use a gradient run using a buffer in the pH range 3.5 to 4.0. The buffer pH 3.5 was found most appropriate for robust resolution of all components of interest in a minimum run time. Finally, the gradient program was optimized by performing trials on the impurity mixture solution and on the forced degraded LER sample preparations. A column oven temperature of 50°C and flow rate of 1 ml/minute were chosen to obtain better chromatography with a shorter run time.

**Fig. 3:**
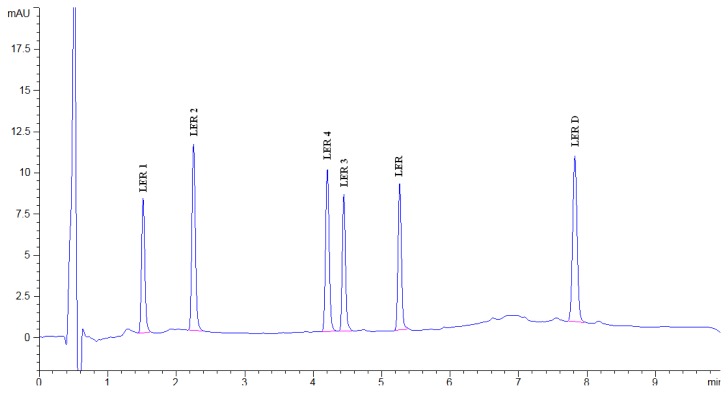
Chromatograph of all separated peaks

LER, its known impurities, and all other unknown degradation impurities were all resolved in the reasonable time of 10 minutes. A typical chromatograph showing the separation of all components is represented in Fig. 3.

### Results for Method Validation

#### System Suitability

The %RSD of the area of three replicate injections was below 2.0%. The tailing factor of all the peaks was in the range of 0.95 to 1.22. Results of the other system suitability parameters are presented in Table 1. From the observed data, the acceptable system suitability parameters would be: relative standard deviation of replicate injections should not be more than 2.0%, theoretical plates should not be less than 2000, tailing factor should not be more than 2.0, and the resolution between LER-3 and LER-4 should not be less than 2.5.

#### Specificity

Overlaid chromatographs of the blank preparation, placebo preparation, impurity mixture preparation, and LER standard preparation were observed, no interference was found on any peak of an impurity as well as on the peak of LER from the blank and placebo. The peak purity factor for each peak was found to be greater than 0.998. It indicated the method’s capability to separate all peaks without any interference.

**Fig. 4:**
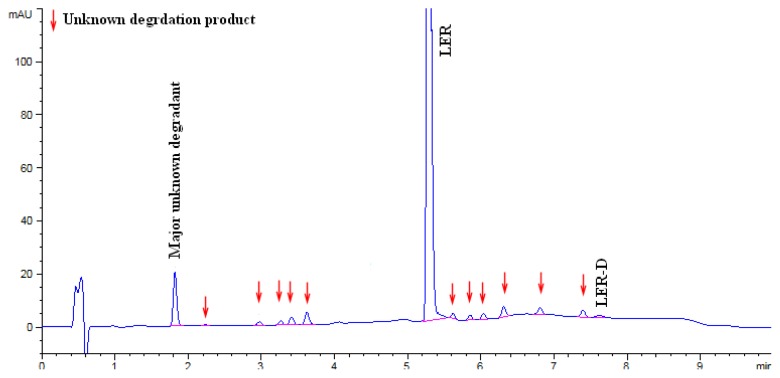
Chromatograph of acid degraded tablet sample

**Fig. 5:**
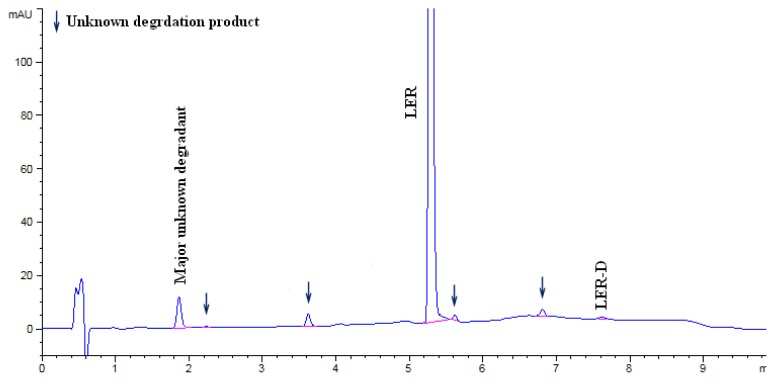
Chromatograph of base degraded tablet sample

**Fig. 6:**
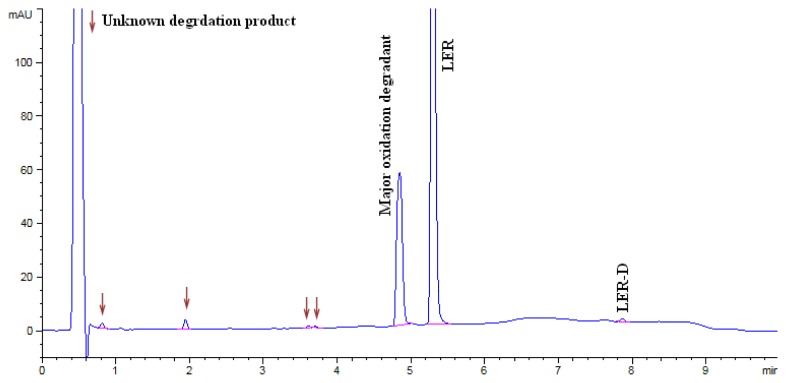
Chromatograph of oxidation degraded tablet sample

**Fig. 7:**
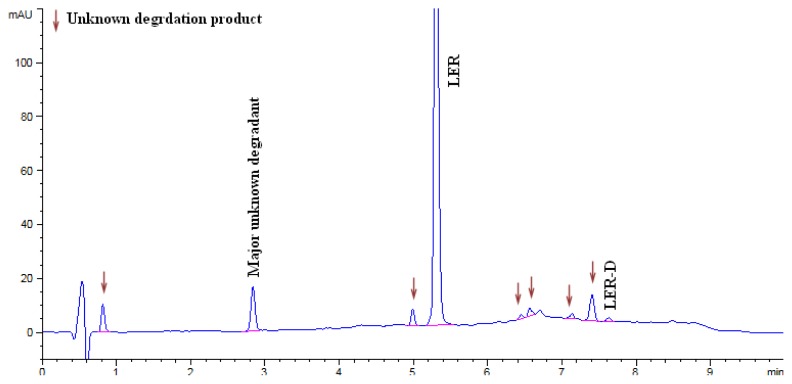
Chromatograph of thermal degraded tablet sample

**Fig. 8:**
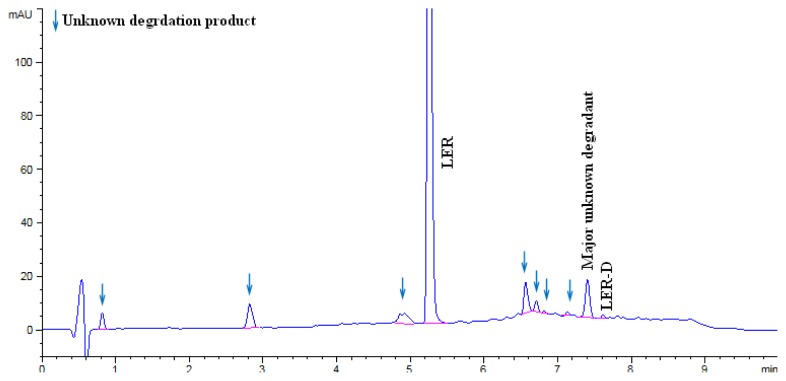
Chromatograph of thermal with moisture degraded tablet sample

**Fig. 9:**
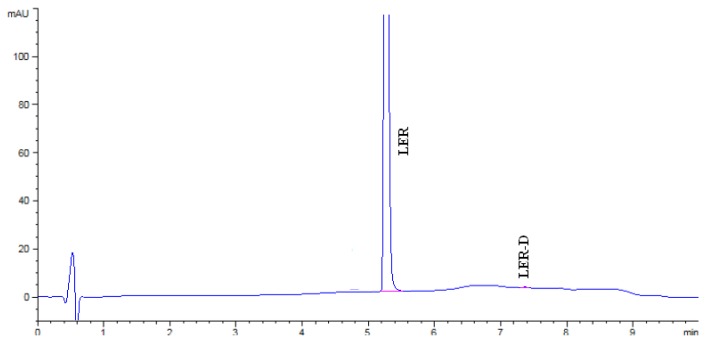
Chromatograph of untreated tablet sample

The LER dropped to 87.16% in acid hydrolysis and the degradation peaks were observed in the chromatogram. An unknown degradation product with an area of 5.1% was observed as a major degradation product at a RRT (relative retention time) of about 0.35. The LER dropped to 93.22% in base hydrolysis and the degradation peaks were observed in the chromatogram. An unknown degradation product with an area of 3.7% was observed as a major degradation product at a RRT of about 0.35. The LER was found to be 74.16% in oxidation degradation and an unknown degradation product with an area of 23.58% was observed as a major degradation product at a RRT of about 0.91. The LER dropped to 83.41% in thermal degradation, and a major unknown degradation product with an area of 5.52% was observed at a RRT of about 0.54. The LER dropped to 74.09% in thermal moisture degradation. The major degradation products of the thermal moisture degradations were two unknown impurities with areas of 7.49% and 5.44% at RRTs of about 1.39 and 1.24, respectively. In photolytic degradation, LER impurity D was a major degradation product with an area of 0.4%. Further, the spectra of all known impurities and unknown degradation products were investigated for spectral purity in the chromatogram for all exposed samples and standards and found to be spectrally pure. The chromatographs of the acid, base, oxidation, thermal, and thermal moisture degraded tablet samples are presented in Fig. 4, Fig. 5, Fig. 6, Fig. 7, and Fig. 8, respectively. The chromatograph of the untreated tablet sample preparation is presented in Fig. 9. The results of the force degradation study are given in Table 3.

#### LOD and LOQ

The concentration (in μg/ml) with a signal-to-noise ratio of at least 3 was considered as the LOD and the concentration with a signal-to-noise ratio of at least 10 was considered as the LOQ. The LOD and LOQ results of LER and its impurities are presented in Table 2.

#### Linearity

For all components, the correlation coefficient was greater than 0.999. Values of the correlation coefficients and slopes of the main compound and of impurities are presented in Table 2. The response factors are also presented in Table 2.

#### Accuracy

The amount recovered was within the acceptance criteria at each level. It indicated that the method is accurate. The results of the recoveries are shown in Table 2.

#### Precision

The %RSD value for each impurity was below 2%. Low %RSD values show good reproducibility and repeatability of the method. The %RSD values obtained in the precision and intermediate precision are presented in Table 2.

#### Robustness

No significant effect was observed on the system suitability parameters such as resolution, tailing factor, theoretical plates, and RSD of the compounds of interest when small, but deliberate changes were made to the chromatographic conditions. In all parameters, the impurities determined in the sample preparation (only LER-D was detected in range from 0.13% to 0.14%) were the same as the impurities determined in the sample preparation in the unchanged conditions (only LER-D was detected with value of 0.14%). Thus, the method was found to be robust with respect to variability in the above conditions. As the retention time of LER-D was highly dependent on the pH of the buffer, a small change in the retention time of LER-D was observed in the robustness parameter, but unchanged in its value.

#### Stability of Sample Solution

Solutions were found to be stable for 24 hours at ambient temperature in light and in the dark. LER-D was found to be 0.14% in the sample preparation stored for 24 hours in light which is the same as the initial sample preparation, though LER-D is a photolytic degradation impurity. Solutions were found to be stable at least for 48 hours at 15°C.

## Applications

The development of the LER tablet must consider the challenge of formulating a bioequivalent drug product of such a drug substance having low bioavailability. Most formulation developers develop their formulation strategies for such a drug product based on the modification of physical properties such as crystal forms, drug substance particle size, and the use of different excipients in variable quantities as binder, disintegrator, diluents, or lubricator like magnesium stearate, sodium starch glycolate, lactose monohydrate, microcrystalline cellulose, mannitol, and others. They also modify the formulation process by using dry or wet granulation of the drug substance with excipients, the top spray technique, and other techniques.

This validated methodology was evaluated successfully during all of these processes of final formulation development and by analyzing various developed formulations with variant formulation strategies in their shelf life period. During the stability study it was observed that the drug product which was packed in a lower pocket size aluminium blister pack was more stable compared to that packed in a higher pocket size aluminium blister pack. In the drug product which was packed in a higher pocket size aluminium blister pack, the value of the LER-D and the value of an unknown impurity which was observed as a major oxidation degradation product (RRT of about 0.91) in the oxidation stress study were obtained in higher amounts.

The proposed method was also applied for the determination of impurities of LER in two other tablet formulations named Zanidip® 20 mg tablet (Solvay Pharma, Thailand) and Lercadip® 10 mg tablet (Zuoz pharma, Venezuela). Impurities of LER were calculated against the LER diluted standard preparation. In Zanidip® and Lercadip®, the amount of LER-D was 0.043% and 0.068%, respectively. The amount of a single maximum unknown impurity was 0.028% and 0.034% in Zanidip® and Lercadip®, respectively. Both tablets showed a single maximum unknown impurity peak just next to the peak of LER-3 and before the peak of LER, which was observed as a major unknown degradation product in the oxidation stress study (RRT of about 0.91).

## Conclusion

A rapid resolution liquid chromatography method was successfully developed and validated for the simultaneous determination of process impurities and degradation impurities of LER in pharmaceutical dosage form. Method validation results have proven that the method is selective, precise, accurate, and robust with stability-indicating power. Furthermore, this paper has presented a shorter method that could be utilized by analytical scientists in the pharmaceutical field instead of using long methods. The low %RSD values indicated the suitability of this method for the routine analysis of the LER dosage form without any interference from the excipients and related impurities. The shorter run time of 10 minutes with better solution stability for at least 48 hours allows the analysis of more than 50 samples per day at a comparatively lower cost.

## Figures and Tables

**Tab. 1:** Gradient program

Time (minutes)	%A, (pH 3.5, 0.01 M phosphate buffer)	%B, (Acetonitrile)
0.0	70	30
5.0	45	55
6.0	30	70
8.0	30	70
8.5	70	30
10.0	70	30

**Tab. 2:** Method validation results for system suitability, linearity, and accuracy

Validation Parameter	LER-1	LER-2	LER-4	LER-3	LER	LER-D
System suitability parameters						
%RSD^a^	0.94	0.61	0.69	0.84	0.76	0.51
Column efficiency	4738	9259	29875	43974	59786	67979
Resolution	–	–	–	3.29	–	–
Linearity (μg/ml)	0.01–3.0	0.01–3.0	0.01–3.0	0.01–3.0	0.01–3.0	0.01–3.0
Correlation coefficient	0.99997	0.9999	0.9999	0.99999	0.9997	0.99995
Slope	13.7513	20.2624	18.2834	13.5479	15.6418	22.5085
Relative response factor^b^	0.879	1.295	1.169	0.866	1.000	1.439
LOD (μg/ml)	0.01	0.01	0.01	0.01	0.01	0.01
LOQ (μg/ml)	0.05	0.03	0.03	0.03	0.05	0.03
%RSD at LOQ^c^	3.4	2.8	4.1	5.3	3.9	2.1
Accuracy, mean % recovery at						
LOQ^d^	90.1	103.6	103.1	102.1	96.4	96.1
50%^e^	101.8	97.8	99.3	98.7	101.3	98.7
100%^e^	98.8	99.6	98.8	101.1	100.4	100.9
150%^e^	100.3	99.1	99.6	100.3	100.2	101.0
Precision						
%RSD (n=6)	1.7	1.4	0.9	1.8	1.1	1.7
Intermediate precision						
%RSD (n=6)	1.1	1.2	1.3	1.7	1.2	1.1

a determined on three replicate injections;

b slope of impurity solution in curve/slope of reference solution in curve;

c determined on six replicate injections;

d considering 0.05% of sample concentration;

e with respect to target specification concentration.

**Tab. 3:** Forced degradation data

Degradation condition	LER	
	
	% Assay	Major degradation products
No degradation (controlled sample)	99.9%	–
Acid hydrolysis (1 N HCl, 80°C, 1 hour)	87.16%	Major unknown impurity (5.1% at RRT 0.35)
Base hydrolysis (1 N NaOH, 80°C, 1 hour)	93.22%	Major unknown impurity (3.7% at RRT 0.35)
Oxidation degradation (10% H_2_O_2_, 50°C,1 hour)	74.16%	Major unknown impurity (23.58% at RRT 0.91)
Thermal degradation (100 °C, 6 hours)	83.41%	Major unknown impurity (5.52% at RRT 0.54)
Thermal moisture degradation (spiked 10% water, 100 °C, 6 hours)	74.09%	Two major unknown impurities (7.49% at RRT 1.39 and 5.44% at RRT 1.24)
Photolytic degradation (1.2 Million lux hours)	99.2%	LER D (0.4%)
